# A Simple Method for Isolating Fucoxanthin, Which Shows a Wide Range of Physiological Effects, from Microalga, *Chaetoceros calcitrans*

**DOI:** 10.3390/molecules31101707

**Published:** 2026-05-18

**Authors:** Akari Numase, Rei Ohtsu, Kiyohiko Suzuki, Yoshinori Kawazoe

**Affiliations:** 1 Graduate School of Advanced Health Sciences, Saga University, Saga 840-8502, Japan; 2 MIDAC Co., Ltd., Hamamatsu 431-3122, Japan; r.ootsu@midac.jp; 3 MIDAC HOLDINGS Co., Ltd., Hamamatsu 431-3122, Japan; 4 Center for Bioresource Education and Research, Saga University, 152-1 Shonan-cho, Karatsu 847-0021, Japan

**Keywords:** fucoxanthin, microalgae, purification, biological activity

## Abstract

Fucoxanthin is a carotenoid belonging to the xanthophyll family and has been shown to exhibit various physiological activities. While brown algae have traditionally been used as a source of fucoxanthin, microalgae have been attracting attention as an alternative source to brown algae in recent years. Although various methods have been devised to isolate fucoxanthin from microalgae, these methods have drawbacks such as requiring special equipment or being unsuitable for large-scale production. Therefore, we tried to develop a simple method that anyone can easily try and that allows for mass production. First, the extraction yields using various solvents were compared. Acetone showed the most efficient extraction yield for short extraction times, but as the extraction time increased, there was almost no difference in the extraction yields among methanol, ethanol, and acetone. Compared to ethanol, methanol had an extraction efficiency of approximately 98%, while acetone had an extraction efficiency of 90%. Then, our efforts have resulted in the development of a method that can isolate fucoxanthin with a purity of over 90% with just a single run of a silica gel column chromatography. Furthermore, the fucoxanthin with this method retained a wide range of physiological activities, including antioxidant, anti-cancer, and anti-inflammatory effects. Our findings might broaden the scope of fucoxanthin research and contribute to process development of fucoxanthin.

## 1. Introduction

**Figure 1:**
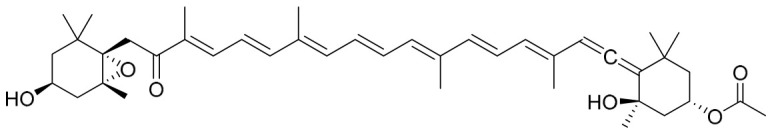
A chemical structure of fucoxanthin.

Fucoxanthin (FX, Figure 1) is a marine xanthophyll carotenoid predominantly found in brown or red algae [1,2,3]. This pigment is responsible for the characteristic brownish color of these organisms and plays an important role in light harvesting during photosynthesis. This natural product, unlike β-carotene or lutein, is characterized by possessing a unique chemical structure, including an allenic bond and an epoxide group (Figure 1). It is believed that these structural features contribute to its potent biological activities [4,5,6,7], such as antioxidant [8,9], anti-inflammatory [10,11], anti-obesity [12,13,14,15], and anti-cancer effects [16,17,18]. As a result, FX has attracted significant attention as a bioactive compound for use in pharmaceuticals, functional foods, dietary supplements, or cosmetic products.

Traditionally, FX has been extracted and purified from macroalgae, particularly brown algae such as *Undaria pinnatifida* and *Saragassun horneri*, as well as certain red algae species [19]. However, the content of FX in these sources is relatively low, and biomass availability is influenced by the season, weather, and environmental conditions. These difficulties prevent macroalgae FX production from scalability and economic feasibility.

To overcome these limitations, microalgae have recently attracted attention as a promising alternative and potentially more sustainable source of FX. Microalgae offer several advantages including rapid growth [20,21], high photosynthetic efficiency [22,23], and the ability for year-round cultivation under controlled conditions [22,23,24]. In addition to this, recent research has revealed the existence of microalgae that can produce and store socially useful substances [25], such as alternative fuels to petroleum [26] or unsaturated fatty acids like eicosapentaenoic acid and docosahexaenoic acid [27], through photosynthesis.

Obtaining natural products from living organisms generally involves two processes: extraction and purification. Similarly, obtaining FX from microalgae also involves these two processes, and various methods have been attempted to achieve satisfactory results. For example, ultrasound-assisted extraction, microwave-assisted extraction, deep eutectic solvents, and enzymes such as pectinase and cellulase have been used in the extraction process [28]. For the purification process, evaporation, solid phase extraction such as a hydrophile–lipophile balance column [29], a high-performance liquid chromatography (HPLC) system [30], or centrifugal partition chromatography [31] have been examined. Furthermore, attempts are being made to perform extraction and purification simultaneously using supercritical carbon dioxide [32]. However, these methods had several disadvantages, including the need for specialized and expensive equipment and skilled operators, low processing capacity, high operating costs, thermal decomposition, and difficulty in optimizing conditions and scaling up.

Under these circumstances, this study attempted to develop a method that allows anyone to easily obtain high-purity FX by combining common, classical, and simple techniques. We selected a marine microalga, *Chaetoceros* sp., as a model microalga for FX production and then established an efficient extraction condition and developed a user-friendly purification method utilizing silica gel column chromatography. Our method enabled us to isolate as high as 92.6%-purity FX with only one column run. In addition, the FX obtained using this method maintained various biological activities, suggesting its potential as a user-friendly method for preparing active FX. The silica gel one-run purification method developed in this study offers exceptional purity of over 92%, significantly simplified purification routes, dramatically reduced introduction, maintenance, and operating costs, contribution to green chemistry, and ease of operation and scale up. Our findings may contribute to the development of a sustainable and efficient production system for FX, supporting its broader utilization in health-related industries.

## 2. Results

### 2.1. Extraction and Isolation of FX from Microalgae

We began this study with the establishment of simple identification and quantification methods of FX. To this end, HPLC analysis was employed, which allows us simultaneous identification and quantification of substances based on retention time and peak area, respectively. Using authentic FX samples, HPLC separation studies were conducted to determine the suitable conditions (see Materials and Methods in detail). In our hands, the retention time of FX under these conditions was approximately 8.1 min (Figure S2). Subsequently, a standard curve was prepared from the peak areas of authentic standard samples of known quantities (Figures S3 and S4 and Table S1). The R-squared value of the calibration curve was 0.9996, suggesting that the curve fit very well.

**Figure 2:**
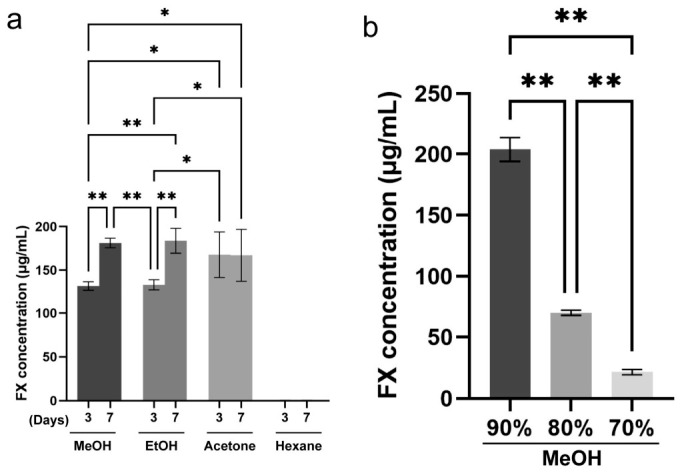
Extraction profile of FX for various solvents or methanol concentrations, where 0.5 g (wet weight) of microalga was extracted with 10 mL of methanol (MeOH), ethanol (EtOH), acetone, or hexane for 3 and 7 days (**a**). Approximately 0.5 g (wet weight) of microalgae was extracted with 10 mL of 90%, 80%, or 70% methanol for 3 days (**b**). The FX content of these crude extracts was quantified by HPLC analysis. For statistical analysis, two-way analysis of variance (**a**) or one-way analysis of variance (**b**) was used. Each value is the mean ± standard error of the mean of three independent determinations. *: *p* < 0.05; **: *p* < 0.01.

Using the determined measurement method stated above, the extraction conditions from the microalga were examined. For this purpose, a method of immersion in an organic solvent was chosen because it is accessible to anyone and easy to scale up. Moreover, instead of using special equipment, we tried to compensate by extending the extraction period. We first compared the differences in extraction efficiency due to different extraction solvents. Approximately 0.5 mg (wet weight) of *Chaetceros calcitrans* CCAP-1085/3 (CCAP: Culture Collection of Algae and Protozoa, Scotland, UK) was soaked in 10 mL of methanol, ethanol, acetone, or hexane, and incubated in a refrigerator for 3 or 7 days. After the indicated period, the extracts were passed through a 0.45 μm membrane filter and subjected to HPLC analysis to examine the extraction efficiency of FX (Figure 2a). Methanol and ethanol showed similar trends in the amount of FX extracted, with the amount increasing with longer extraction times. On the other hand, acetone extracted a greater amount than methanol or ethanol at 3 days of extraction; however, extending the extraction period showed little effect. Interestingly, FX was hardly extracted with hexane from microalgae. When the FX extraction amount in long-term extraction with ethanol was set to 100, the extraction efficiency of methanol under the same conditions was 98 and that of acetone was 90. Considering the above results and the cost of each solvent, we decided to use methanol for the following experiments.

Next, the methanol concentration dependency of FX extraction was investigated. FX was extracted from 0.5 g of wet microalga using 10 mL of 90%, 80%, or 70% of methanol for 3 days, and the amount of extracted FX was measured by HPLC analysis. As shown in Figure 2b, the lower the methanol concentration, the less FX was extracted. It was noteworthy that 90% methanol had a better extraction efficiency than methanol. Therefore, we decided to carry out subsequent experiments using the extraction protocol of “90% methanol, for 3 days” (see Materials and Methods in detail).

Having determined the extraction conditions, we next tried to establish a simple purification method. Analyses of 90% methanol crude extract by thin-layer chromatography (TLC) indicated that, in addition to FX, the extract contained at least chlorophyll a and chlorophyll c. To obtain high purity FX, we attempted to purify FX using silica gel column chromatography. Based on the TLC results, a 1:3 mixture of hexane and ethyl acetate was chosen for the developing solvent, and the characteristic color of FX was used as an indicator for separation. After collecting FX, the identity and purity of the obtained FX was examined. As a result, although FX was identified (Figure S5, upper panel), its purity was less than 10%. Based on the total ion chromatogram (TIC; electrospray ionization positive mode) of liquid chromatography–tandem mass spectrometry (LC-MS/MS) analysis, there were several peaks other than FX that were observed (Figure S5, lower panel). Among these, some peaks eluted earlier than FX could not be identified but might be degradation products derived from carotenoids or chlorophyll (Figure S6). On the other hand, the four peaks that eluted later than FX were presumed to be a type of triglyceride (Figure S7). To remove triglyceride contamination, the composition of the developing solvent used in the silica gel column separation was changed. We devised a two-step elution method, which involved first washing away lipids with a low-polarity solvent, then purifying FX by increasing the polarity of the developing solvent. More specifically, lipids were eluted with a mixture of a hexane: ethyl acetate ratio of 3:1 (FX stayed at the origin point under this condition), and then FX was eluted with a developing solvent of higher polarity (hexane: ethyl acetate = 1:1).

**Figure 3:**
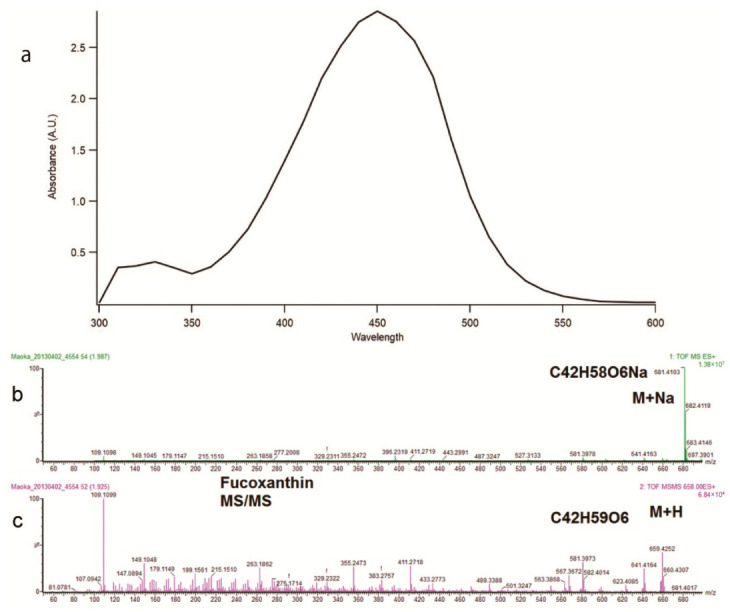
Spectroscopic analysis of purified FX. Purified FX was analyzed by ultraviolet–visible absorption (**a**), liquid chromatography–mass (**b**), and liquid chromatography–tandem mass (**c**) spectroscopic analysis.

To demonstrate whether scaling up is possible or not, we attempted to purify FX on a larger scale with the method developed here. Using approximately 100 g of wet algal cells as a starting material yielded approximately 160–200 mg of FX. And the purity of the FX was 79.47 ± 3.86% (shown as mean ± standard deviation, *n* = 3). These results suggested that our efforts to purify FX from microalga had established a method for isolating FX with a high degree of purity by only one column run. In addition to quantitative tests, qualitative analyses of purified FX were carried out. The ultraviolet–visible absorption spectrum was almost identical to that of the standard sample, with the maximum absorption wavelength at 450 nm (compare Figure 3a with Figure S1). As a result of high-resolution mass spectrometry, the parent ion peak was observed at *m*/*z* 681.4103 (for [M + Na]^+^), suggesting that the molecular formula was C_42_H_58_O_6_ (calcd 681.4126), which is identical to that of FX (Figure 3b). Furthermore, the fragmented ions observed in LC-MS/MS (Figure 3c) were also identical to those previously reported for FX: the parent ion *m*/*z* 659.4 was transited to daughter ions *m*/*z* 641.4, 581.4, and 109.1 [33,34]. The results of all the measurements shown here were consistent with those of FX, strongly suggesting that the substance purified was FX.

Next, we tried to improve our method to obtain even higher purity FX. Until then, we had been performing separations that balanced the purity and yield. Here, we adopted a strategy that sacrificed yield in order to prioritize purity. Specifically, during column purification, the beginning and end of the FX release were ignored and only the central portion was harvested. As a result, although the yield did decrease (143.20 ± 6.5 mg from wet weight 100 g of microalga, shown as mean ± standard deviation, *n* = 3), the purity improved dramatically and became almost equivalent to that of HPLC purification (purity of 92.62 ± 5.6%, shown as mean ± standard deviation, *n* = 3). The purified product was confirmed to be FX, as the ^1^H nuclear magnetic resonance (NMR) spectra (Figure S8) showed a perfect match with the previous literature [35]. Furthermore, ^1^H NMR analysis revealed that only trace-level impurity peaks, presumably originating from methyl and methylene groups, were detected, which was consistent with the high purity.

### 2.2. Bioactivities of FX Derived from Microalga

FX has been shown to have various physiological effects, including antioxidant, anti-cancer, anti-inflammatory, and anti-obesity effects. However, natural products often undergo chemical changes such as decomposition, oxidation, polymerization, or isomerization during extraction or purification processes and lose their physiological functions. Since FX is a compound highly sensitive to heat, light, and oxygen, it was necessary to confirm whether the FX purified by this method retained its physiological activity or not. A highly versatile 80% purified product was used as the sample at the test tube or cultured cell levels. In the following experiments, FX concentrations were adjusted based on the amount of FX contained.

#### 2.2.1. Antioxidant Activity

To assess antioxidant activity, two of the most widely used methodologies, 2,2-diphenyl-1-picrylhydrazyl (DPPH) and oxygen radical absorbance capacity (ORAC) assays, were performed.

DPPH assay measures the electron-transfer capacity of antioxidants using the stable DPPH radical. It is favored for its rapidity and cost-effectiveness; however, it is limited by low physiological relevance. In contrast, the ORAC assay assesses the hydrogen atom transfer capacity against a biologically relevant peroxy radical, providing a better reflection of in vivo activity. In this study, we used two evaluation methods to examine the antioxidant capacity of FX in terms of Trolox per unit amount.

**Figure 4:**
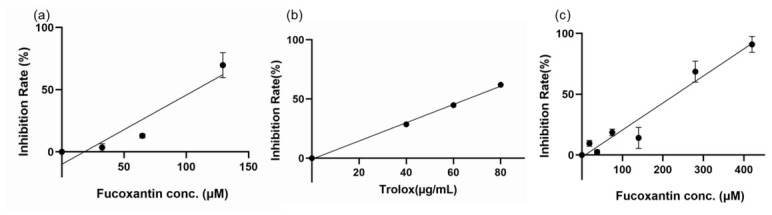
Evaluation of antioxidant activity by DPPH method. Radical scavenging activity of FX (**a**), Trolox (**b**), and HPLC-purified FX (**c**). Each value is the mean ± standard error of the mean of three independent determinations.

In the DPPH assay, the radical scavenging activity of FX was increased in a concentration-dependent manner (Figure 4a). The IC_50_ value of FX was 110.80 μM (72.56 μg/mL) and that of Trolox (Figure 4b) was 69.61 μg/mL. Thus, the Trolox-Equivalent Antioxidant Capacity (TEAC) of FX measured by the DPPH method was calculated as 0.96 mgTE/mg. On the other hand, the Trolox value measured using the ORAC method was 7.88 ± 1.90 mgTE/mg. FX showed strong absorption at 450 nm; it may have interfered with the DPPH method measurement, resulting in a difference between the two, even though the absorption of FX alone was subtracted.

#### 2.2.2. Anti-Cancer Activity

**Figure 5:**
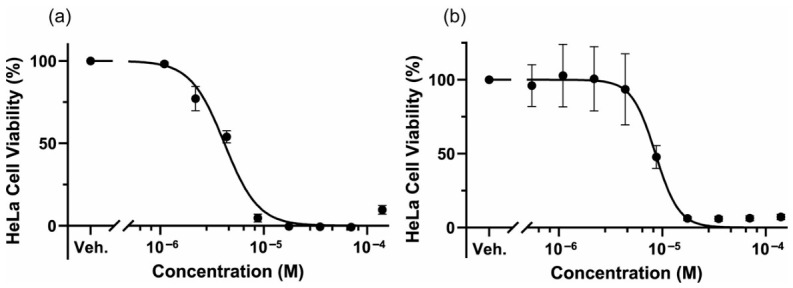
Inhibitory effect of FX against cancer cell proliferation. HeLa cells were incubated in the presence of various concentrations of 80% purity FX (**a**) and HPLC-purified FX (**b**) for 48 h, then cell growth was examined using WST-8 reagent. Each value is the mean ± standard error of the mean of three independent determinations.

HeLa cells seeded onto 96 well plate were incubated in the presence or absence of FX for 48 h, then cell viability was evaluated with water soluble tetrazolium salt (WST)-8 reagent (Figure 5a). There was no effect on low concentrations, but FX showed a strong inhibitory effect of HeLa cell growth at higher concentrations. The IC_50_ value was calculated to be 4.16 μM.

#### 2.2.3. Anti-Inflammatory Activity

To confirm that microalga-derived FX showed anti-inflammatory activity, the lipopolysaccharide (LPS)-induced tumor necrosis factor (TNF)-α levels in the absence or presence of FX were measured. The human monocytic cell line THP-1 was differentiated into macrophage by phorbol 12-myristate 13-acetate (PMA) and stimulated with LPS with various concentrations of FX for 3 days. Then, TNF-α concentrations in the supernatant were measured by enzyme-linked immunosorbent assay (ELISA). As shown in Figure 5a, LPS-induced TNF-α levels were reduced in an FX concentration-dependent manner: 80.0, 60.0, and 38.9% at 1.25, 2.5, and 5.0 μM FX, respectively. Simultaneously, cell viability was also examined using the WST-8 reagent. Almost no effect was observed at low concentrations; however, cell viability decreased to 95.0, 86.9, and 54.5% at 1.25, 2.5, and 5.0 μM FX, as seen in HeLa cells (Figure 5b). Therefore, the reduced level of TNF-α at 5.0 μM might be due to cell death rather than an anti-inflammatory effect.

#### 2.2.4. Anti-Diabetic Activity

**Figure 6:**
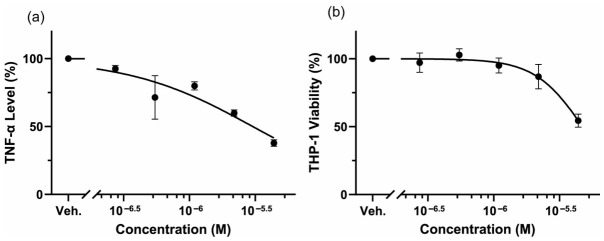
Anti-inflammatory effect of FX. THP-1 cells were differentiated into macrophages and stimulated by LPS in the presence or absence of FX for 72 h. Then, culture medium was collected and evaluated the TNF-α level by ELISA (**a**). Simultaneously, cell viability was examined by WST-8 reagent (**b**). Each value is the mean ± standard error of the mean of three independent determinations.

Fully differentiated 3T3-L1 adipocytes were incubated with or without FX for 24 h and stimulated with insulin for 30 min. Then, glucose uptake was measured with a florescence-labeled glucose analog. However, no significant effects were observed throughout the concentration ranges examined (Figure 6a).

### 2.3. The Physiological Activity of Highly Purified FX

The above experiments investigated the physiological activities of approximately 80% purity FX obtained by a single column run purification. However, these experiments could not rule out the possibility that the bioactivities obtained were the results of the action of impurities. Therefore, we used highly purified FX obtained by HPLC to perform some of the physiological activity tests mentioned above. The FX with a purity of 80% obtained by silica gel column chromatography was further purified by HPLC to obtain FX with a purity of 98.5%. Using this highly purified FX, DPPH antioxidant, tumor cell proliferation inhibition, and glucose uptake tests were performed.

**Figure 7:**
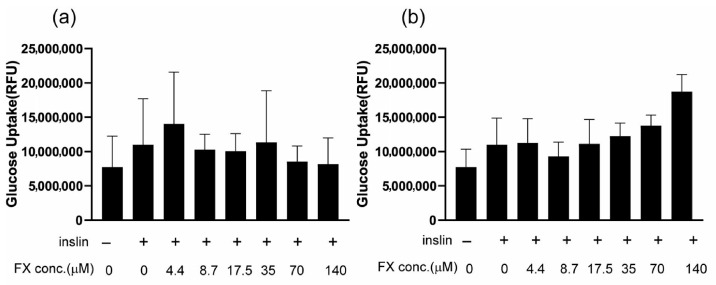
Anti-diabetic activity of FX. 3T3-L1 cells were induced differentiation into fat cells and incubated with various concentrations of 80–purity FX (**a**) or HPLC-purified FX (**b**) for 16 h. Then, the cells were stimulated with insulin for 30 min, and glucose uptake activity was examined. Each value is the mean ± standard error of the mean of three independent determinations. Two-way analysis of variance was applied, but no significant difference was observed.

First, the DPPH antioxidant capacity was 0.50 mgTE/mg (Figure 4c), which was close to the value for 80% purity FX (0.96 mgTE/mg). Next, the dose–response curve for HeLa cell proliferation (Figure 5b) was very similar to that of the 80% purity FX (Figure 5a). At this time, the IC_50_ value was 3.71 μM, which also showed good agreement with the 80% purity FX (4.16 μM). Finally, its effect on glucose uptake was examined. No effect was observed at low concentrations, but as the concentration of FX increased, glucose uptake tended to be promoted, although the difference was not statistically significant (Figure 7b).

## 3. Discussion

*Chaetoceros calcitrans* is a marine microalga belonging to the diatom family and has been used as feed for farmed bivalve shellfish. Diatoms and brown algae photosystem contain a light-harvesting pigment protein named fucoxanthin chlorophyll-binding protein [36,37]. This protein contains pigment molecules called chlorophyll c and FX, which are not found in terrestrial plants. In this study, we successfully established the user-friendly isolation system of FX with more than 90% purity from the *Chaetoceros calcitrans* CCAP-1085/3 strain by only one column run. In addition to purity, the yield was also excellent: the purified yield normalized to biomass reached approximately 7.15 mg/gDW (we obtained approximately 140 mg of FX from 100 g wet weight of *Chaetoceros* sp.; 100 g wet weight is approximately equivalent to 20 g dry weight). Furthermore, we confirmed that the FX purified using this method retains the various physiological effects of FX that have been reported to date. The purification method established in this study is user-friendly and does not require special equipment, which makes it accessible to anyone and potentially encourages participation in FX research. Moreover, since it is considered easy to apply large-scale preparations, it is expected to contribute to the sustainable and stable supply of FX.

A previous study has shown that ethanol was the most efficient solvent for extracting FX from the diatom *Phaeodactylum tricornutum*, and ethanol at a 70% concentration was the most effective when used [38]. On the other hand, the extraction efficiencies for methanol and ethanol were almost the same in *Chaetoseros* sp. (Figure 2b). Although *Chaetoceros* and *Phaeodactylum* are both diatoms, they belong to different genera. Therefore, the structure of the frustule is likely different even though the main component is the same silica. This is thought to be the reason for the difference in FX extraction efficiency from the two diatoms. Consequently, it would be beneficial to consider different extraction solvents depending on the microalgae species, even within the same diatom family. In addition to this, cost-effective methanol was selected, but the use of less-toxic ethanol would be recommended if considering the application of FX for supplements.

Sun et al. extracted diatoms with 70% ethanol and then used an evaporator to evaporate the solvent to obtain a specific ethanol concentration, thereby obtaining FX with a purity of approximately 80% [38]. Another study successfully obtained FX with a purity of 91% by combining open octa decyl silyl column chromatography with evaporative enrichment [39]. Furthermore, purification by HPLC yields an FX of >99% [40]. It is true that these methods have succeeded in obtaining high-purity FX, but the use of evaporators or HPLC seems unsuitable for large-scale production. On the other hand, our two-step elution method using silica gel is easily scaled up and can obtain as high as 92% pure FX in a single operation. Therefore, our developed method is considered more suitable for the mass production of FX from large-scale cultured microalgae, because it offers a purity of over 90%, simplified and cost-effective purification system, and contribution to green chemistry.

On the other hand, while this method is very simple, there are many things that need to be confirmed at each step for mass production, such as methods for removing microalgae residue from the extraction process (they pass through ordinary filter paper), methods for removing large amounts of solvent while preventing the decomposition of FX, and/or whether simply scaling up lab-scale experiments at the same rate is sufficient for operating columns at mass production levels of 100 L or more. However, now that the problems have become clear, there is a high possibility of mass production when optimizing each step.

Considering versatility during large-scale preparation, we deliberately used FX with a purity of 80% for physiological activity tests. As a result, it became clear that even FX with a purity of 80% exhibited a wide range of biological activities. However, we could not rule out the possibility that the remaining 20% of impurities, rather than FX, might exhibit physiological activities or that impurities might have adverse effects on living organisms. Therefore, we used a more highly purified form of FX, specifically FX with a purity of 98.5%, obtained through HPLC, to investigate selected physiological activities. We examine the antioxidant, anti-cancer, and insulin-mediated glucose uptake effects. The results showed good agreement between the 80% purity and 98.5% purity samples regarding antioxidant and anti-cancer effects. However, regarding glucose uptake on adipocytes, 80% purity FX did not show any effects (Figure 7a), whereas 98.5% purity FX exhibited a tendency to promote glucose uptake at higher concentrations, though the difference was not significant (Figure 7b). These results suggested that even FX with 80% purity can be effectively applied depending on how it is used. Since it does not always exhibit sufficient bioactivity, high purification and selective use are considered necessary depending on the desired physiological activities.

Previous studies have reported that the IC_50_ values of FX for HeLa cell proliferation were 1445 μM [41] or 55.1 μM [42]. However, our own results show similar values of 4.1 μM or 3.7 μM, although there are slight differences, probably due to variations in FX purity. Thus, even for the same IC_50_ value for HeLa cells, there is a difference of approximately 400 times. Since the treatment period was the same in all reports (48 h), these differences may be due to cell density or differences in FX batches.

To our knowledge, there are no reports that clearly calculate the IC_50_ value for antioxidant capacity testing using the DPPH method. While there was a report of 79.6–111.2 μg/mL using crude extracts of brown algae, it indicated that FX was present in the crude extract, but its content was not mentioned [43]. Furthermore, there were few studies that have used the ORAC method to determine the TEAC value for the antioxidant activity of FX. Therefore, this was the example that determined the IC_50_ or TEAC value for the antioxidant capacity of FX.

To date, the anti-inflammatory effects of bioactive molecules have been investigated using the suppression of pro-inflammatory cytokines or nitric oxide production as indicators. Regarding the anti-inflammatory effects of FX, Lee et al. reported that FX inhibits the LPS-induced induction of pro-inflammatory cytokines, interleukin-1β, interleukin-6, and TNF-α production through blocking NF-κB activation using the mouse macrophage cell line RAW246.7 and mouse bone marrow-derived macrophages [44]. In addition, Zhang et al. also reported that FX inhibited TNF-α production in response to LPS stimulation in a concentration-dependent manner using human monocytic cell line THP-1 cells differentiated into macrophages [45]. Our results were consistent with these previous studies, suggesting that FX might block TNF-α production in macrophages. However, none of these reports have calculated the IC_50_ value. This might be due to the cytotoxicity at high concentrations of FX.

Previous studies have reported that FX does not promote glucose uptake in 3T3-L1 adipocytes [46]. In our study, a slight promoting trend was observed. This discrepancy may be due to differences in FX concentration: we treated the cells at 10 times the concentration used in previous studies. In any case, it did not appear that FX significantly promotes glucose uptake in adipocytes. On the other hand, FX has been shown to lower blood glucose levels in the mouse experiments [47,48]. Taken together, FX might primarily act on tissues other than adipose, such as muscles, to exert its blood sugar-lowering effect.

While various studies have been conducted on the physiological functions of FX to date, each study focused on a single bioactivity and used different FX lots. In contrast, as far as we know, this study is the first example that conducts a wide range of bioactivity tests using the same lot of FX from an identifiable origin. Therefore, this study might serve as a common guideline for future research on the physiological functions of FX.

## 4. Materials and Methods

### 4.1. Reagents and Instruments

Commercially available FX (Wako Pure Chemical, Osaka, Japan) was used as an authentic standard. All solvents used in this study were extra-pure grade and purchased from Nacalai Tesque (Kyoto, Japan). Silica gel Chromatorex PSQ 100B (Fuji Silysia Chemical, Kasugai, Japan) and Silica gel 60 F_254_ (Merck, Darmstadt, Germany) were used for column chromatography and TLC, respectively. Spectroscopic data were obtained by SpectraMax iD3 multimode plate reader (Molecular Devices, San Jose, CA, USA). Mass and LC-MS/MS analysis were performed with Acquity LC Xevo G2-S Q Tof MS (Waters, Milford, MA, USA) in the electrospray ionization positive ion mode. NMR spectrum was recorded on Varian INOVA-500 (Burker, Billerica, MA, USA).

### 4.2. Microalga Strain and Culturing Conditions

For strain selection, we purchased eight microalgal strains (one of which lives in freshwater and the rest in seawater) from Culture Collection of Algae and Protozoa (CCAP, Scotland, UK). After comprehensively considering the ease of cultivation, maintenance costs, and recovery rate in the event of contamination, we decided to use the *Chaetoceros calcitrans* CCAP-1085/3 strain for further experiments.

Artificial seawater was prepared with Marine Art SF-1 (Tomita Pharmaceutical Co., Ltd., Tokushima, Japan) according to the manufacturer’s instructions. Then, 9.0 g of metasilicate nonahydrate (WAKO Pure Chemical, Osaka, Japan) and 100 mL of complex amino acid solution (Daiichi Co., Ltd., Kumamoto, Japan) were added separately per 100 L of artificial seawater. Aeration was performed at a rate of 8.0 L per minute using a gas mixture containing carbon dioxide to achieve a concentration of 5.0% in the indoor air relative to artificial seawater.

A small-scale precultured CCAP1085/3 strain was cultured in 500 mL of seawater with stirring at 350 rpm for 3 weeks to prepare a seed strain. For the main cultivation, 4 L of the seed strain was added to 100 L of artificial seawater, and the cultivation was carried out under continuous illumination with LED light for 24 h with aeration. The microalgae were monitored daily using appearance, cell number, and turbidity (ABS = 680 nm). The cultivation was completed when the cell density reached approximately 1 × 10^7^ cells/mL.

ThermoFisher LYNX4000 centrifuge (Waltham, MA, USA) was used for harvesting microalgae. Centrifugation was performed at 9000 rpm for 5 min at room temperature. For 100 L culture, centrifugation was conducted over two days. The harvested microalgae were immediately frozen and kept at −70 °C until use.

### 4.3. Identification and Quantification of FX

To identify and quantify the FX in the sample simultaneously, we employed an HPLC analysis. A reversed-phase column (COSMOSIL 5C_18_-AR-II, 4.6 I.D. × 150 mm, Nacalai Tesque) was used, and elution was performed using a linear gradient of acetonitrile in H_2_O, starting with 80% of acetonitrile and increasing to 100% of acetonitrile over 15 min. FX was detected by the absorbance at 450 nm from a preliminary absorption spectrum measurement of the authentic sample (Figure S1). Under these conditions, the retention time of FX was approximately 8.1 min (Figure S2). A standard curve was prepared from the peak areas of FX in the HPLC chromatograms of several authentic FX samples with known absolute amounts (Figures S3 and S4), and the FX in the *Chaetoceros calcitrans* samples were quantified.

### 4.4. Extraction, Quantity Evaluation, and Purification of FX from the Microalga

For the identification and quantification, I0.5 g (wet weight) of *Chaetceros* sp. was soaked in 10 mL of extraction solvent and incubated in a freezer for 3 or 7 days. The crude extracts were passed through a 0.45 μm membrane filter to remove insoluble materials and subjected to HPLC analysis. For the purification of FX, approximately 100 g (wet weight) of *Chaetceros* sp. (approximately 20 g dry weight) was immersed in 2 L of 90% methanol, subjected to an ultrasonic cleaning machine for 10 min and incubated in a freezer for 3 days. After that, residue was removed by filtration to obtain crude extracts. The crude extracts were allowed to evaporate the solvent by rotary evaporator, and the resulting extracts were subjected to silica gel column chromatography. After loading the crude extract onto the column (50 × 150 mm for crude extract obtained from 100 g wet weight of alga), two column volumes of hexane: ethyl acetate (3:1) were run through the column, and then the developing solvent was switched to a 1:1 mixture of hexane and ethyl acetate. FX was recovered using its characteristic color as an indicator.

### 4.5. Physiological Activities of FX

#### 4.5.1. Anti-Cancer Cell Proliferation Assay

Human cervical cancer cell line HeLa cells were cultured in Dulbecco’s Modified Eagle Medium (DMEM) supplemented with 10% fetal bovine serum (FBS). For the viability assay, the cells were seeded onto a 96 well plate at a density of 1 × 10^4^ cells/well. After incubating for 48 h with various concentrations of FX, cell viability was measured by using Cell Counting Kit-8 (WST-8 reagent, DOJINDO, Kumamoto, Japan) according to the manufacturer’s instruction. WST-8 is a tetrazolium salt. When added to a cell culture, it is reduced to form an orange-colored product called formazan. Just before adding WST-8 reagent, the cells were washed with phosphate-buffered saline (PBS) three times to remove FX, which interfered with WST-8 reagent.

#### 4.5.2. Antioxidative Effect of FX

The antioxidant effect of FX was investigated using two different methods: the DPPH and ORAC method.

When an antioxidant donates a hydrogen atom to the purple-colored DPPH radical, it becomes the non-radical, non-colored DPPH-H. This change in color is measured spectrophotometrically at 517 nm, where a decrease in absorbance indicates higher antioxidant activity. DPPH stock solution was prepared by dissolving DPPH reagent (DOJINDO, Kumamoto, Japan) in ethanol at a concentration of 10 mg/mL. A total of 100 μL of reaction mixture (10 μL of FX solution in methanol, 40 μL of PBS, and 50 μL of DPPH stock solution) was put into a 96 well plate, incubated for 30 min at room temperature in darkness, and absorbance was measured at 517 nm.

The ORAC assay measures antioxidant inhibition of peroxyl radical-induced oxidation of a fluorescent probe, fluorescein. As antioxidants scavenge the radicals, they delay the decay of the fluorescence signal over time. The antioxidant capacity is then quantified by calculating the area under the curve (AUC) relative to a standard, such as Trolox. ORAC method was performed by means of H-ORAC Activity Assay Kit (Wako Pure Chemical) according to the manufacture’s instruction.

#### 4.5.3. Anti-Inflammatory Effect

Human monocytic cell line THP-1 was maintained in RPMI-1640 medium supplemented with 10% FBS. The cells in the 96 well plate at a density of 1 × 10^4^ cells/well were incubated with 200 ng/mL of PMA for 48 h, then stimulated with LPS for 72 h. TNF-α level in the culture supernatant was determined by means of Human TNF-α DuoSet ELISA (R&D Systems, Minneapolis, MN, USA) according to the manufacture’s instruction.

#### 4.5.4. Anti-Diabetic Effect

Mouse fibroblastic cell line 3T3-L1 was cultured in the DMEM supplemented with 10% adult calf serum. The cells were induced adipogenesis by stimulating DMEM containing 10% FBS, 5 μg/mL of insulin, 0.5 mM of dexamethasone, and 0.5 mM of 3-isobutyl-1-methylxanthine for 2 days, then 10% FBS and 5 μg/mL of insulin for another 2 days. After that, the culture medium was replaced with fresh DMEM containing 10% FBS every 2 days. After 10 days of induction, fully differentiated 3T3-L1 adipocytes were incubated with several concentrations of FX for 24 h. Then, the cells were stimulated with 5 μg/mL of insulin in glucose-free DMEM for 30 min, and glucose uptake was evaluated by glucose uptake assay kit (DOJINDO, Kumamoto, Japan) according to the manufacture’s instruction.

### 4.6. Statistical Analysis

Statistical analyses were processed by GraphPad Prism 10 software (Dotmatics, Boston, MA, USA). Comparison of mean values was performed using one-way or two-way analysis of variance to confirm significance, and then Tukey’s test was used for multiple comparisons.

## 5. Conclusions

In this paper, a method for obtaining FX from the microalga *Chaetoceros* sp. was developed. This method combined simple and classical techniques and can be performed by anyone. Specifically, it combined ethanol extraction of the alga with silica gel column chromatography purification. Despite its simplicity, it can produce FX with a purity of over 90%. Furthermore, the FX obtained by this method retained a wide range of biological activities. Because this method provides a simple and cost-effective purification system, it is expected to be applicable to the mass production of FX using microalgae.

## Data Availability

The data presented in this study are available on request from the corresponding author.

## Supplementary Materials

The following supporting information can be downloaded at: https://www.mdpi.com/article/10.3390/molecules31101707/s1, Figure S1: Ultraviolet–visible spectrum of authentic fucoxanthin sample; Figure S2: HPLC chromatogram of authentic fucoxanthin sample; Figure S3: HPLC analysis of authentic fucoxanthin samples with known absolute amounts for creating a standard curve; Figure S4: Standard curve for fucoxanthin quantification; Figure S5: HPLC/PDA/MS analysis of purified FX; Figure S6: LC-MS/MS analysis of peaks eluted earlier than FX; Figure S7: LC-MS/MS analysis of impurities thought to be of lipid origin; Figure S8: NMR spectra of purified FX; and Table S1: Peak area determined by HPLC for Figure S4.
