# Supplementary material for: A Simple Method for Isolating Fucoxanthin, Which Shows a Wide Range of Physiological Effects, from Microalga, Chaetoceros calcitrans
Source: Molecules. 2026 May 18;31(10):1707. doi: 10.3390/molecules31101707 (PMC13209740; doi:10.3390/molecules31101707)
Supplement: Supplementary file 1 [file molecules-31-01707-s001.zip › molecules-4300003-supplementary.pdf]

Supplementary Materials

A Simple Method for Isolating Fucoxanthin, Which Shows a Wide Range of Physiological Effects, from Microalga, *Chaetoceros calcitrans*

Akari Numase [1], Rei Ohtsu [2], Kiyohiko Suzuki [3] and Yoshinori Kawazoe [1,4,*]

[1] Graduate School of Advanced Health Sciences, Saga University, Saga 840-8502, Japan
[2] MIDAC Co., Ltd., Hamamatsu 431-3122, Japan; r.ootsu@midac.jp
[3] MIDAC HOLDINGS Co., Ltd., Hamamatsu 431-3122, Japan
[4] Center for Bioresource Education and Research, Saga University, 152-1 Shonan-cho, Karatsu 847-0021, Japan
* Correspondence: ykawazoe@cc.saga-u.ac.jp; Tel.: +81-955-77-4484

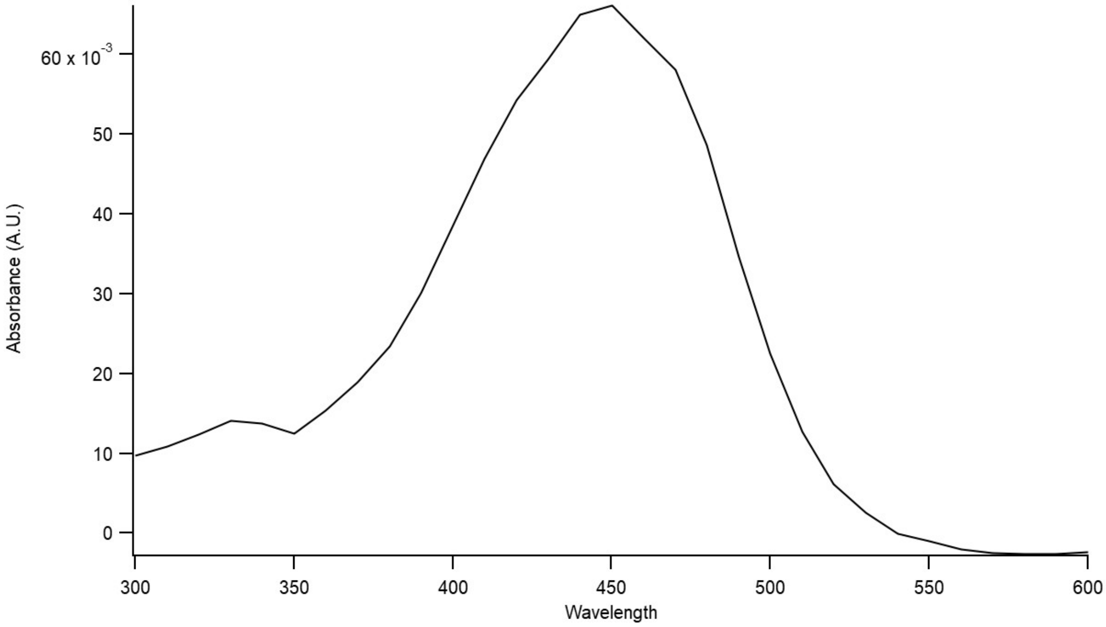

Figure S1. Ultraviolet-Visible spectrum of authentic fucoxanthin sample

The maximum absorption wavelength was 450 nm.

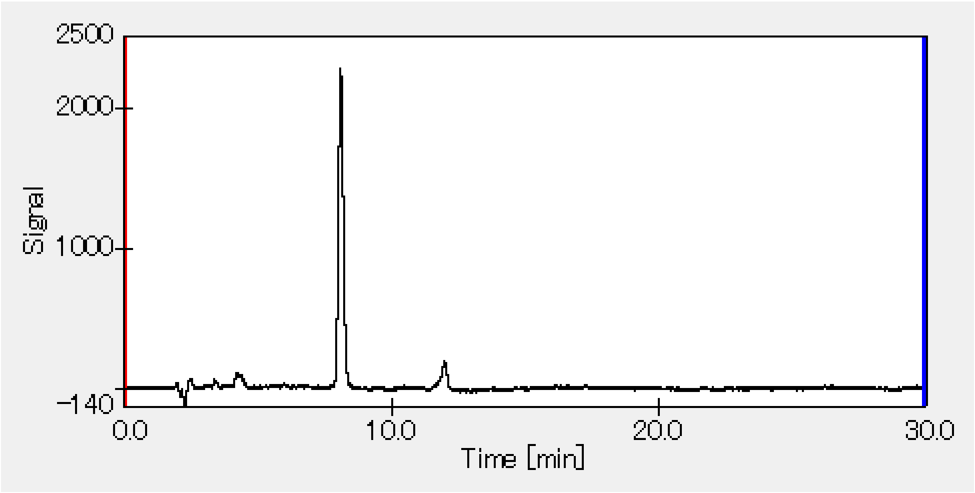

Figure S2. HPLC chromatogram of authentic fucoxanthin sample

HPLC analysis was performed using a linear gradient from 80% to 100% acetonitrile in H_2O over 15 minutes, under which the retention time of fucoxanthin was 8.1 minutes.

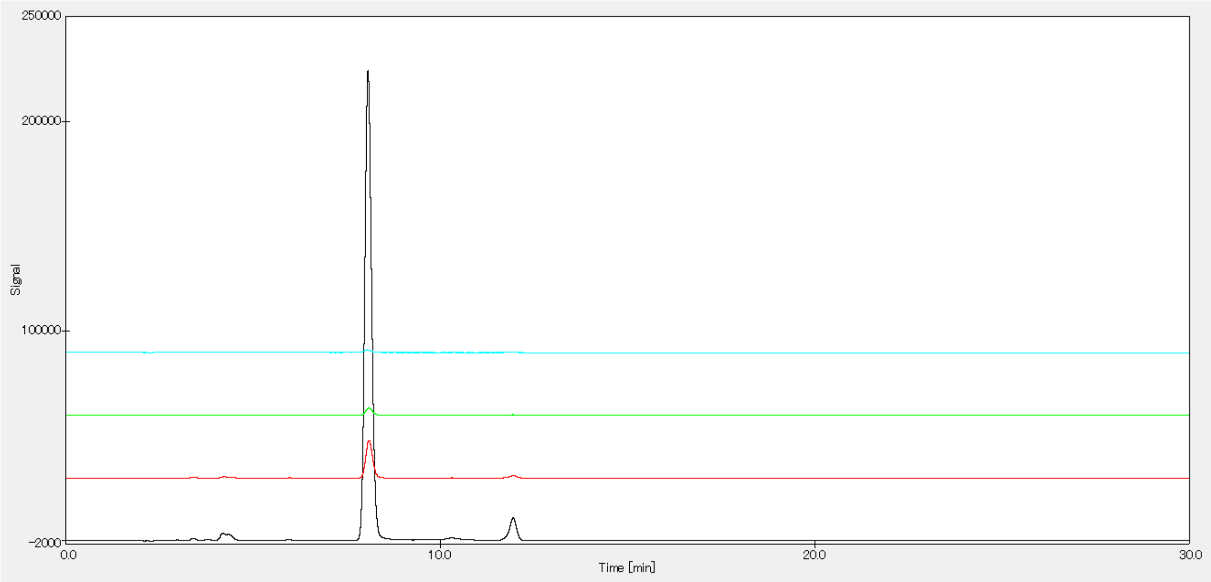

Figure S3. HPLC analysis of authentic fucoxanthin samples with known absolute amounts for creating a standard curve

1,000 ng (black), 100 ng (red), 20 ng (green), and 10 ng (pale blue) of authentic fucoxanthin samples were analyzed by HPLC under the same conditions as in Figure S2.

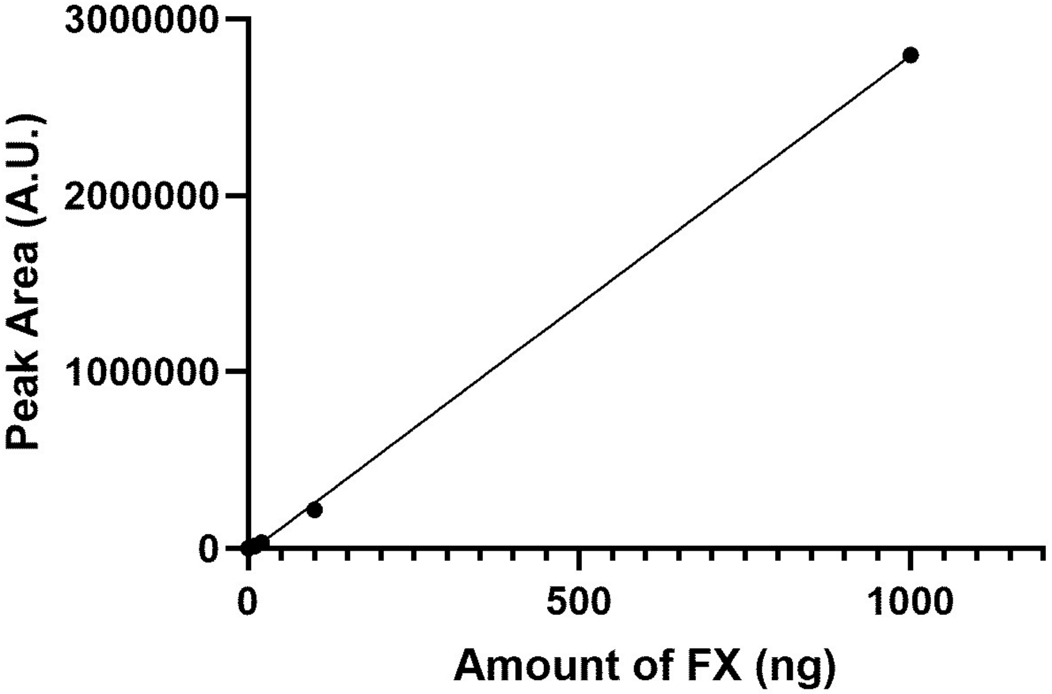

Figure S4. Standard curve for fucoxanthin quantification

In the HPLC chromatogram of Figure S3, the fucoxanthin peak area (See Table S1) was plotted against the amount of fucoxanthin to create a standard curve. The determination coefficient was 0.9996.

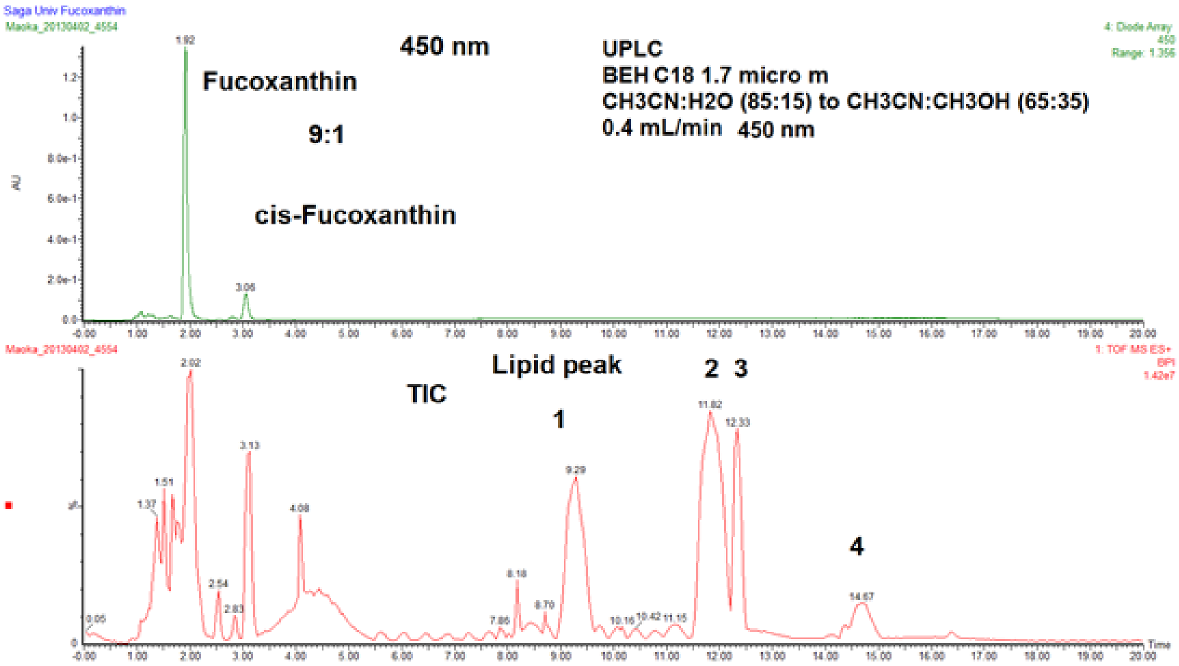

Figure S5. HPLC and total ion chromatography analysis of purified FX in first trial Purified FX in first trial was analyzed by HPLC detected at 450 nm absorbance (upper panel) and total ion chromatography (Electrospray Ionization Positive mode) of LC-MS/MS analysis (lower panel).

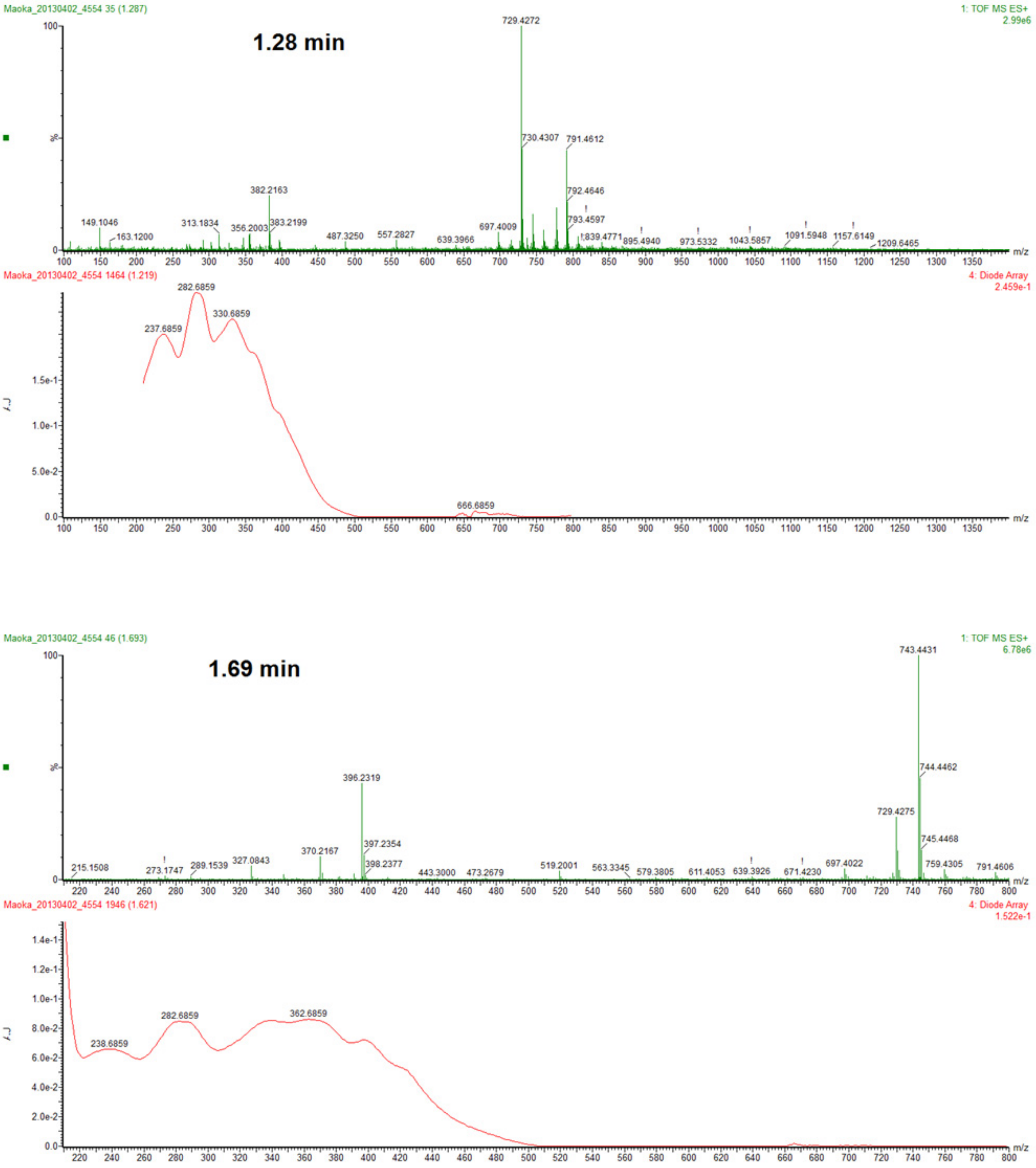

Figure S6. LC-MS/MS analysis of peaks eluted earlier than FX

LC-MS/MS and UV/VIS chromatogram of the retention time of 1.28 min peak (upper panels) and those of 1.68 min peak (lower panel)

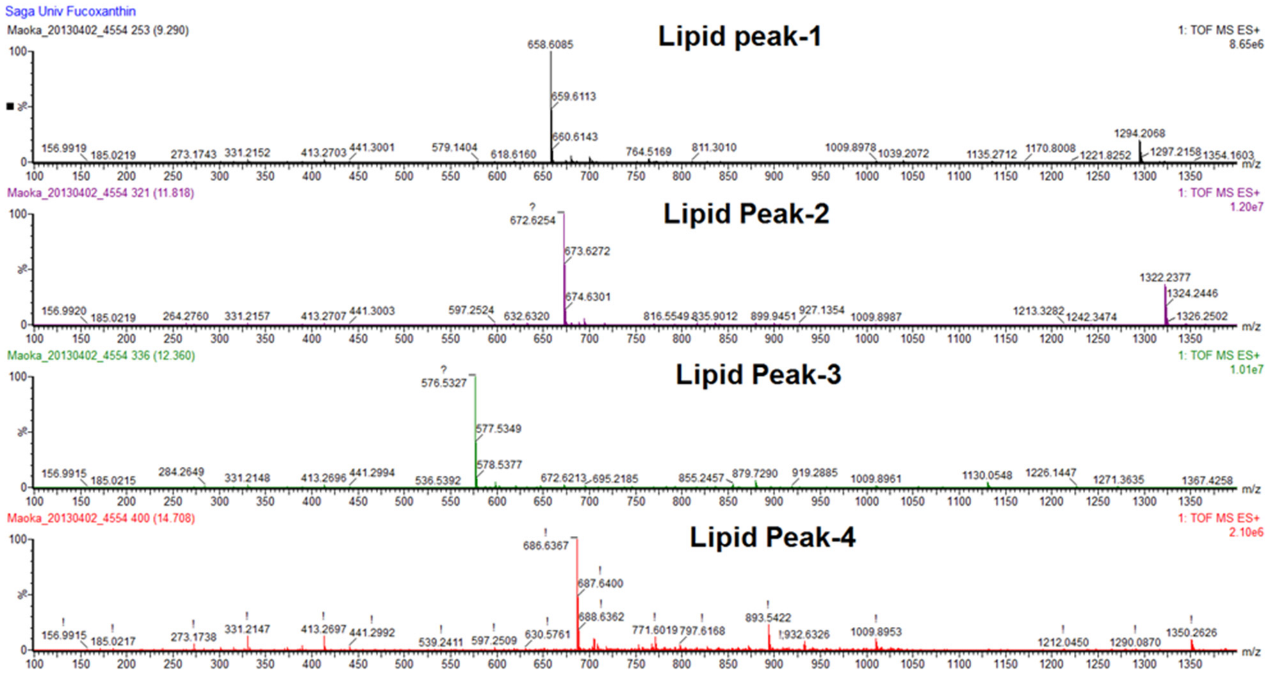

Figure S7. LC-MS/MS analysis of impurities thought to be of lipid origin

LC-MS/MS chromatograms for the four impurity peaks (see Figure S5).

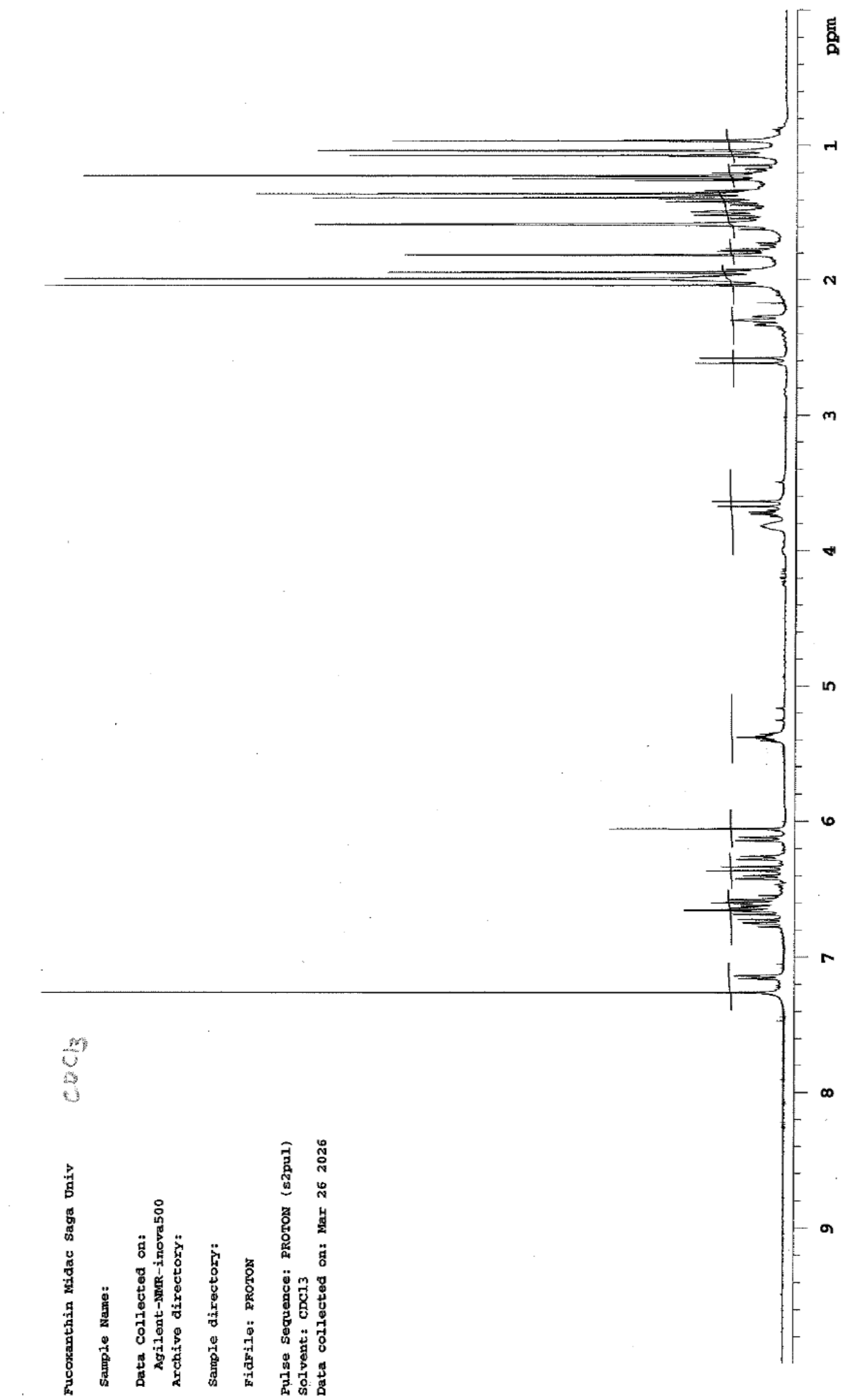

Figure S8. 1H-NMR spectrum of silica gel-purified FX (92% purity)

500 MHz, $CDCl_3$

Table S1. Peak area determined by HPLC for Figure S4

Amount of FX (ng)	Peak area
0	0
10	13822.6
20	34000.3
100	218305.6
1000	2797048.4
